# An intervention study of the general practice management of older adults with cardiovascular metabolic comorbidities

**DOI:** 10.1371/journal.pone.0350895

**Published:** 2026-06-24

**Authors:** Yunjing Li, Zhehua Zou, Shuangyan Yi, Yue Yang, Guanli Yuan, Jingping Xu

**Affiliations:** General Medical Department, The First Hospital of Qinhuangdao, Qinhuangdao, Hebei, China; Kurume University School of Medicine, JAPAN

## Abstract

**Purpose:**

To investigate the impact of general practice management on treatment compliance, lifestyle, and clinical indicators in older adults with cardiovascular metabolic comorbidities to provide more effective disease management strategies for this population.

**Patients and methods:**

This randomized controlled trial enrolled 200 older adults with type 2 diabetes and established atherosclerotic cardiovascular disease (e.g., coronary heart disease, ischemic stroke) who were discharged from Qinhuangdao First Hospital between November 2021 and May 2023. Patients were randomly divided into intervention and control groups (n = 100 per group). The intervention group received general medical management, including personalized health education during hospitalization, community outpatient follow-up after discharge, and lifestyle interventions. The control group received routine discharge guidance. Changes in medication usage, body mass index (BMI), smoking rate, and clinical indicators, such as glycated hemoglobin, low-density lipoprotein cholesterol, and creatinine levels were evaluated over a 12-month follow-up period. Dietary intake and physical activity were assessed using the Semi-Quantitative Food Frequency Survey Questionnaire and the Physical Activity Scale for the Elderly, respectively.

**Results:**

After 12 months, 91 controls and 95 intervention patients remained and were analyzed per-protocol. Within-group analyses revealed that the intervention group successfully maintained high utilization rates of all recommended medications (metformin, SGLT2i, GLP-1RA, aspirin/clopidogrel, statins, ACEI/ARB), whereas the control group experienced significant declines (all P < 0.05). Both groups achieved significant reductions in BMI, but only the intervention group showed a significant decrease in smoking rates (P = 0.014). The intervention group demonstrated comprehensive dietary improvements (e.g., increased nuts, dairy, vegetables, and aquatic products; reduced refined carbohydrates, meats, and salt) and significantly increased physical activity scores (all P < 0.05). Clinically, while both groups achieved reductions in glycated hemoglobin and LDL-C, only the intervention group showed significant improvements in creatinine levels, whereas renal function in the control group significantly deteriorated (P < 0.001).

**Conclusion:**

A structured general practice management model effectively sustains long-term medication adherence, promotes comprehensive healthy lifestyle modifications, and optimizes key metabolic and renal clinical indicators in older adults with cardiovascular metabolic comorbidities.

**Trial registration:**

Chinese Clinical Trial Registry ChiCTR2500112212.

## Introduction

Chronic comorbidities represent a prevalent health concern in older adults (defined in the cited review as those aged ≥ 60 years), with a global prevalence rate of 37.2% [1]. Its incidence rate increases significantly with age, reaching 58.1% in China [2]. Such conditions seriously impact the physical health and overall quality of life of older adults. Cardiovascular metabolic comorbidities refer to multiple concurrent cardiovascular and metabolic diseases in an individual, including but not limited to sugar metabolism diseases (type 2 diabetes), stroke, lipid metabolism diseases (hypercholesterolemia or hypertriglyceridemia), primary hypertension, coronary heart disease, etc [3]. According to reports, the incidence rate of cardiovascular metabolic complications in China is 11.6–16.9% [4], while in the United States it is 14.4%, with higher rates among men and the elderly [5]. The disease pattern is relatively stable, with a mortality risk 3.7–6.9 times higher than that of patients without cardiac metabolic diseases, making it one of the most common chronic comorbidities today [6]. Due to the complexity of the factors contributing to the onset of the disease and individual differences among patients, the uncertainty surrounding the prevention and treatment of cardiovascular and metabolic comorbidities far exceeds that of treating a single disease [7]. Therefore, identifying and assessing risk factors for cardiovascular metabolic complications can help optimize the prevention and treatment of cardiovascular metabolic complications and their risk factors.

Recent large-scale population studies in China have identified a close comorbid relationship between cardiovascular disease and abnormal glucose metabolism, further increasing the risk of cardiovascular disease [8,9]. However, previous research studies have highlighted substantial deficiencies in the standardized management of cardiovascular risk for such patients. Specifically, international research from Europe as well as studies in China have shown that the usage rates of hypoglycemic drugs, lipid-lowering agents, and aspirin remain generally low [10,11]. This gap in effective management has contributed to adverse cardiovascular outcomes. The therapeutic success of cardiovascular metabolic comorbidities is closely linked to treatment adherence, in which effective disease management is crucial throughout the process of care. Older adults with cardiovascular metabolic comorbidities often face multiple challenges, including a high burden of simultaneous illnesses (e.g., progressive renal impairment, physical frailty), which can be compounded by factors that may lead to challenges in social support, such as a portion of patients living alone and significant socioeconomic burdens. Collaborative management between general practitioners from tertiary hospitals and community health service centers can alleviate such burdens by reducing the frequency of cross-departmental visits and minimizing risks associated with repeated clinical examinations and inappropriate medication. By improving lifestyle interventions, such as medical nutrition therapy and exercise management, cardiovascular health and the prognosis of these patients can be improved significantly.

In this study, we investigated the impact of general practice management on the enhancement of treatment compliance, correcting unhealthy lifestyles, and alleviating clinical symptoms in older adults with cardiovascular metabolic comorbidities. Ultimately, this study determined a disease management approach tailored to China’s national conditions that is both practical and effective. These findings will help to inform future strategies for the management of cardiovascular metabolic comorbidities, providing guidance for improving patient care and outcomes.

## Materials and methods

### Research objective

In this study, we employed a randomized controlled clinical research designto enroll 200 older adults with specific cardiovascular metabolic comorbidities, defined as meeting the diagnostic criteria for both type 2 diabetes and at least one atherosclerotic cardiovascular disease (ASCVD), such as coronary atherosclerotic heart disease or atherosclerotic ischemic stroke. Patients were recruited using a sequential selection method upon discharge from the General Medical Department of Qinhuangdao First Hospital between 18/11/2021 and 31/05/2023. The inclusion criteria were as follows: (1) compliance with the Guidelines for the Prevention and Treatment of Type 2 Diabetes in China (2020) [12] and meeting the diagnostic criteria for type 2 diabetes; (2) the presence of at least one of the following atherosclerotic cardiovascular diseases (ASCVDs): coronary atherosclerotic heart disease, atherosclerotic ischemic stroke, or peripheral artery disease, based on the diagnostic criteria outlined in Internal Medicine (8th Edition) [13]; and (3) aged ≥ 65 years. The exclusion criteria were as follows: (1) patients with cognitive impairment; (2) patients with advanced malignant tumors; (3) patients unwilling to participate in the study or refusing to undergo follow-up; and (4) patients unable to communicate or interact normally. Furthermore, patients who declined further participation or experienced adverse events during the study, such as accidents or death, were excluded. Patients were randomly allocated (1:1 ratio) into either an intervention or a control group (100 patients per group). The randomization sequence was generated by an independent statistician using SPSS 25.0 software. Allocation concealment was maintained using sequentially numbered, opaque, sealed envelopes, which were opened by the recruiting researchers only after the patient had signed the informed consent form. All participants were adequately informed of the nature and objectives of the study and signed an informed consent form prior to participation. The study was approved by the Ethics Committee of Qinhuangdao First Hospital (2021Q093) and registered with the Chinese Clinical Trial Registry (ChiCTR2500112212). The trial was registered retrospectively on November 11, 2025 (ChiCTR2500112212) due to an administrative oversight. However, we emphasize that the study was conducted strictly in accordance with the protocol approved by the Ethics Committee of Qinhuangdao First Hospital in October 2021. Furthermore, before recruitment began on November 18, 2021, all participants were fully informed of the detailed study protocol, including interventions and potential risks, through written informed consent, ensuring complete transparency prior to enrollment.

### Blinding

Due to the active nature of the health education and community management interventions, blinding of participants and care providers was not feasible. However, to minimize observer bias, all outcome assessors were strictly blinded to the group assignments. These assessors included the laboratory technicians and the research assistants who administered questionnaires and conducted physical examinations.

### Intervention methods

#### Intervention group.

(1) During hospitalization, patients underwent a one-on-one and in-person educational session delivered by senior attending or associate chief physicians from the General Medical Department, alongside clinical dietitians. This session included the distribution of comprehensive health education materials co-authored by general practitioners and nutrition experts. These materials covered various essential topics, including dietary management, exercise routines, weight control, tobacco cessation, blood sugar regulation, the prevention of hypoglycemia, blood pressure and lipid management, medication guidance, the recognition and prevention of emergency situations, and to seek support, if required.
(2) After patients were discharged, the community health service center nearest to their long-term residential address assumed responsibility for providing outpatient follow-up care and self-management training services. These follow-ups were conducted by community general practitioners and nurses who received standardized training from our hospital’s research team. An initial telephone follow-up was conducted within one week of discharge to remind patients to visit the community clinic for their initial follow-up appointment at two weeks post-discharge. For patients with stable conditions, community outpatient follow-ups were scheduled monthly, with laboratory evaluations (specifically, glycated hemoglobin, low-density lipoprotein cholesterol, and creatinine levels) conducted every three months at the community clinic. If a patient’s condition changed, or medication adjustments were required, weekly outpatient follow-up visits were arranged, along with additional community outpatient visits, as required by the patient’s specific condition. If necessary, patients were referred back to the hospital for inpatient treatment. If a patient failed to attend their scheduled community outpatient follow-up, they were promptly contacted by telephone and urged to complete their follow-up appointment as soon as possible.
(3) The general practitioners at our hospital adhered to the Guidelines for the Prevention and Treatment of Type 2 Diabetes in China (2020 Edition) [12], the Chinese Expert Consensus on Comprehensive Management of Cardiovascular Risk in Patients With Cardiovascular Disease Combined With Abnormal Glucose Metabolism [14], and the Guidelines for Preventing Cardiovascular Metabolic Diseases Through Healthy Lifestyle in China [15]. These guidelines provided the foundation for our approach to patient care. To minimize potential intra-clinic correlation and ensure uniform care quality across different community health service centers, all community doctors and nurses involved in this study received uniform, structured training from our hospital’s general practice research team prior to patient enrollment. Following this standardized protocol, our community clinics provided a highly consistent range of services, including the management of medication, lifestyle guidance and patient counseling. Furthermore, every patient visit was meticulously documented, with updates made to the patient’s personal health record (community nurse) to ensure continuity and accuracy in their medical history.

#### Control group.

Upon discharge, patients in the control group received standard discharge instructions (Includes how to take medications after discharge from the hospital, including the name of the medication, the dosage, and the number of times the medication should be taken, and includes a recommendation that the patient go to the hospital outpatient clinic for a follow-up exam in 2 weeks.), and the specified intervention was not implemented.

### Clinical data collection

Before initiating intervention, a comprehensive patient profile was created. Baseline data were collected, including details on current treatment medications, smoking habits, dietary intake, physical activity levels, and key clinical indicators, such as body mass index (BMI), glycated hemoglobin, low-density lipoprotein cholesterol (LDL-C), and creatinine levels.

### Outcome measures

The primary outcomes of this study were defined a priori as the changes in low-density lipoprotein cholesterol (LDL-C) and glycated hemoglobin (HbA1c) levels from baseline to the 12-month follow-up. Secondary outcomes included changes in dietary intake, smoking status, body mass index (BMI), medication utilization rates, physical activity levels, and creatinine levels.

### Laboratory testing methods

All laboratory tests were performed at the clinical laboratory of Qinhuangdao First Hospital. Plasma samples (3 mL) were collected from patients and centrifuged for 8 minutes at 1610 × g. Glycated hemoglobin levels were determined by high-performance liquid chromatography. LDL-C concentrations were determined using a catalase-scavenging method. Creatinine levels were measured using the creatine oxidase method.

### Evaluation tools

All patients were required to attend a clinical follow-up every 12 months. Medication adherence was evaluated using a combination of self-reported structured interviews and objective verification. Specifically, community doctors cross-referenced patient self-reports with electronic prescription refill records in the community health information system. During this evaluation, physical activity levels, dietary habits, and other relevant factors were also systematically reviewed. Furthermore, lifestyle and overall adherence were corroborated by monitoring objective improvements in clinical biomarkers (e.g., HbA1c and lipid profiles) during the follow-up visits.

#### Assessment of physical activity status.

All patients were assessed with the Physical Activity Scale for the Elderly (PASE) [16]. Originally developed by Washburn et al. in 1993, this scale was later translated into Chinese and validated for reliability and accuracy [17]. The PASE scale encompassed three primary dimensions of activities: six items related to leisure activities (e.g., sedentary activities, outdoor walking, light exercise, moderate exercise, high-intensity exercise and muscle endurance training), six items related to household activities (e.g., light household chores, heavy household tasks, home maintenance, lawn mowing, outdoor gardening and caregiving), and one item related to work-related activities (focusing on tasks involving standing or walking, such as paid work or volunteer service). The PASE scale comprised a total of 13 items, with sedentary activities excluded from the calculation used to generate the total score, where a higher score indicates a greater level of physical activity. The remaining 12 items were scored by multiplying the ‘weekly activity frequency’ by the ‘daily activity duration’; then, the product was multiplied by the corresponding weights. The final step involved the summation of scores from all items to generate an overall scale score. A higher total score indicated a greater level of physical activity.

#### Dietary assessment.

To evaluate dietary habits, the Semi-Quantitative Food Frequency Questionnaire [18] was used to determine the frequency and mean intake of various foods consumed by patients over the previous year. Food classification was conducted following the Guidelines for Preventing Cardiovascular Metabolic Diseases Through Healthy Lifestyle in China [15]. Specifically, items were grouped based on similarities in their nutritional composition. The categories included grains and potatoes (e.g., rice and its products, flour-based products, other grains, potatoes, beans, and soy products), nuts, milk and dairy products, vegetables, fruits, meats (including both livestock and poultry meats), internal organs, aquatic products, eggs, edible oils and salt. The mean daily intake for each food category was determined by multiplying the frequency of daily consumption by the mean intake amount per occurrence.

### Ethical considerations

This study was conducted in accordance with the principles of the Declaration of Helsinki. The study protocol was reviewed and approved by the Ethics Committee of The First Hospital of Qinhuangdao (Approval No. 2021Q093). All participants were informed about the study’s purpose and procedures, and all provided written informed consent before enrolment. The study was conducted strictly in accordance with the protocol. However, secondary outcomes regarding sleep quality (PSQI), self-management ability (Modified Diabetes Self-Care Behavior Scale), and quality of life (e.g., SF-36) described in the original protocol are not reported in the current manuscript. Given the complexity and sheer volume of the data presented here (encompassing detailed dietary habits, physical activity scores, medication adherence, and multiple metabolic indicators), including the quality of life analysis would make the paper overly lengthy and dilute its primary clinical focus. Therefore, these psychosocial and functional outcomes will be comprehensively reported in a subsequent, dedicated manuscript.

### Statistical methods

All data were analyzed using the SPSS version 25.0 statistical software package (IBM Corp., Armonk, NY, USA). The Shapiro-Wilk test was used to assess the normality of data distribution. Baseline variables including the intake of beans and products, nuts, fruits, eggs, internal organs, aquatic products, edible oil, salt, leisure activities, work-related activities, LDL-C, and creatinine did not follow a normal distribution (P < 0.05). Consequently, non-parametric Mann-Whitney U tests were used for group comparisons of these variables, and the data were expressed as medians (with 25th and 75th percentiles). Continuous variables that were normally distributed were analyzed using independent t-tests and expressed as mean ± standard deviation. Group comparisons of means were performed using the t-test, while comparisons of medians were conducted using the rank-sum test. The comparison of composition ratios and drug utilization rates across patient groups was performed using the four-cell chi-squared test. A P-value < 0.05 was considered statistically significant.

Data analysis was performed based on a Per-Protocol (PP) approach, including only the 186 patients who completed the 12-month follow-up and provided all primary outcome data. An Intention-to-Treat (ITT) approach with data imputation for dropouts was not employed. The primary outcomes heavily relied on objective biological markers (e.g., HbA1c, LDL-C), and traditional imputation methods could introduce unscientific assumptions.

## Results

### Baseline data

**Fig 1:**
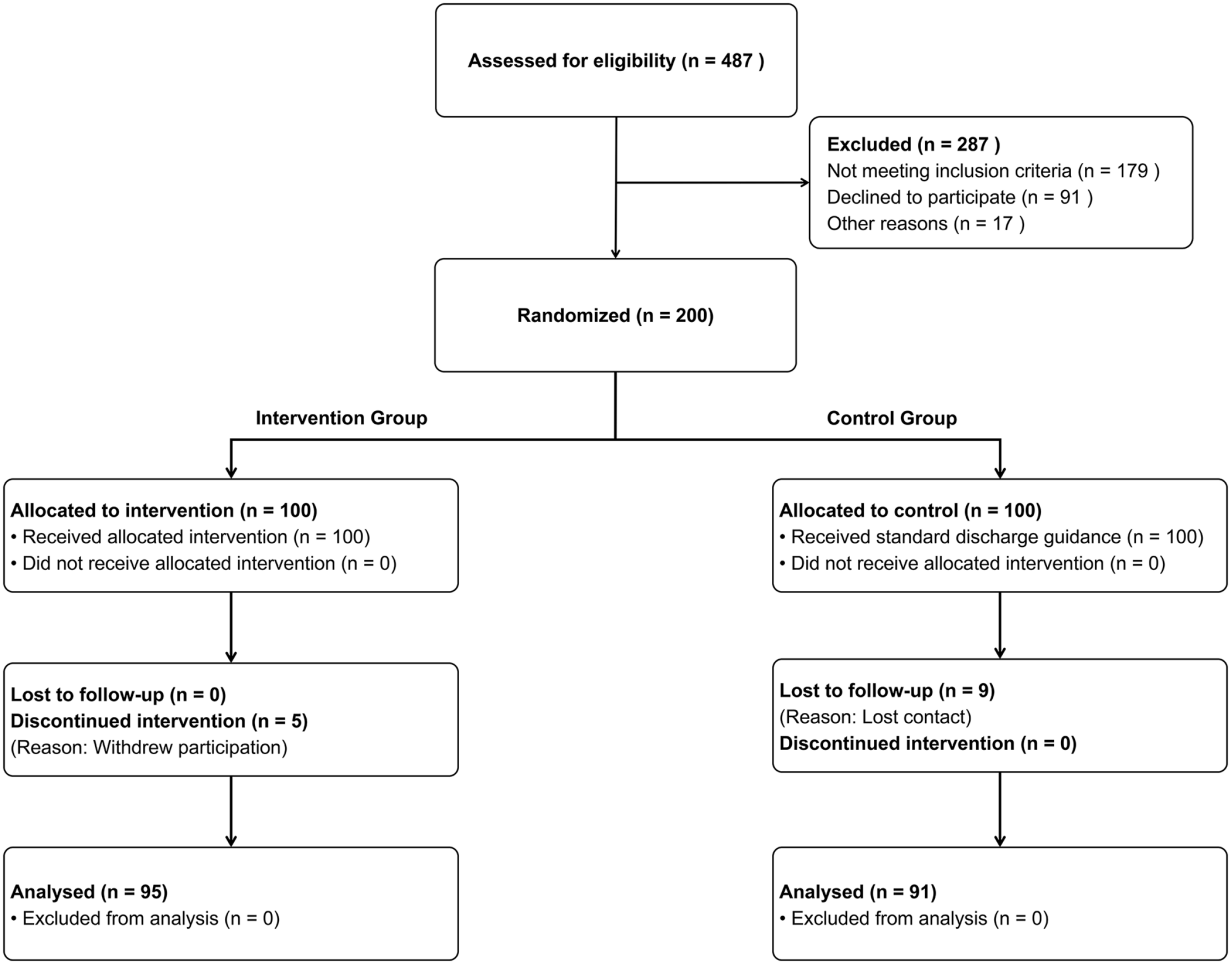
CONSORT 2010 flow diagram. The flow chart depicts the enrollment, randomization, intervention allocation, follow-up, and data analysis of the study participants.

The flow of participants through the study is shown in Fig 1. A total of 200 patients were randomized. During the 12-month follow-up, nine patients in the control group (9.0%) were lost to follow-up after discharge, and five patients in the intervention group (5.0%) withdrew participation (Fig 1). As shown in Table 1, we re-analyzed the baseline demographic characteristics exclusively for the 186 individuals who completed the 12-month follow-up (91 in the control group and 95 in the intervention group). There were still no statistically significant differences between the two groups at baseline in any of the measured variables, including age, sex, educational level, and living arrangements (P > 0.05 for all), indicating that the attrition did not introduce any baseline imbalance.

**Table 1:** Comparison of baseline characteristics between the two groups among the completers.

Characteristics	Control group (n = 91)	Intervention group (n = 95)	Statistic (χ²/ t)	P value
**Sex (Male/ Female)**	54/ 37	51/ 44	χ² = 0.605	0.437
**Age (years)**	71.9 ± 5.1	71.2 ± 4.2	*t* = 0.980	0.328
**Educational level, n (%)**			χ² = 1.302	0.729
Elementary school and below	8 (8.8)	11 (11.6)		
Junior high school	31 (34.1)	37 (38.9)		
High school or vocational school	35 (38.5)	30 (31.6)		
College degree or above	17 (18.7)	17 (17.9)		
**Living alone, n (%)**	8 (8.8)	5 (5.3)	χ² = 0.890	0.345

Notes: Normally distributed continuous variables are expressed as mean ± standard deviation and analyzed using independent t-tests. Categorical variables are expressed as n (%) and analyzed using Chi-square tests.

### Medication use

Analyzing the within-group changes revealed that the medication utilization rates (metformin, SGLT2i, GLP-1RA, aspirin/clopidogrel, statins, and ACEI/ARB) in the control group significantly decreased after the 12-month follow-up (all P < 0.05). In contrast, the intervention group successfully maintained high utilization rates for all recommended medications, with no significant decline observed from baseline to post-intervention (all P > 0.05; Table 2).

**Table 2:** Comparison of medication usage rates before and after intervention within the two groups.

Medication	Group	Before intervention, n (%)	After intervention, n (%)	χ²value	P value
**Metformin**	Control (n = 91)	60 (65.9)	40 (44.0)	8.878	0.003
	Intervention (n = 95)	63 (66.3)	67 (70.5)	0.39	0.532
**SGLT2i**	Control (n = 91)	66 (72.5)	41 (45.1)	14.174	< 0.001
	Intervention (n = 95)	61 (64.2)	58 (61.1)	0.202	0.653
**GLP-1RA**	Control (n = 91)	25 (27.5)	13 (14.3)	4.789	0.029
	Intervention (n = 95)	21 (22.1)	27 (28.4)	1.004	0.316
**Aspirin/ Clopidogrel**	Control (n = 91)	86 (94.5)	75 (82.4)	6.513	0.011
	Intervention (n = 95)	88 (92.6)	89 (93.7)	0.083	0.774
**Statins**	Control (n = 91)	90 (98.9)	72 (79.1)	18.2	< 0.001
	Intervention (n = 95)	92 (96.8)	90 (94.7)	0.13	0.718
**ACEI/ ARB**	Control (n = 91)	63 (69.2)	46 (50.5)	6.61	0.01
	Intervention (n = 95)	71 (74.7)	66 (69.5)	0.654	0.419

Abbreviations: SGLT2i, sodium-glucose cotransporter 2 inhibitor; GLP-1RA, glucagon-like peptide-1 receptor agonist; ACEI, angiotensin-converting enzyme inhibitor; ARB, angiotensin II receptor blocker.

### BMI and smoking status

Following the 12-month period, both groups experienced a significant within-group reduction in BMI compared to their respective baselines (both P < 0.001). Regarding smoking status, the intervention group demonstrated a significant decrease in the number of smokers (from 17.9% to 6.3%, P = 0.014), whereas the control group showed no significant change in smoking rates (P = 0.715; Table 3).

**Table 3:** Comparison of BMI, smoking rate before and after intervention between the two groups.

Variable	Group	Before intervention	After intervention	Statistic (t/χ²)	P value
**BMI (kg/m²)**	Control (n = 91)	25.3 ± 3.2	24.9 ± 3.1	*t* = 4.297	< 0.001
	Intervention (n = 95)	25.2 ± 2.8	23.5 ± 2.1	*t* = 9.430	< 0.001
**Smoking, n (%)**	Control (n = 91)	20 (22.0)	18 (19.8)	χ² = 0.133	0.715
	Intervention (n = 95)	17 (17.9)	6 (6.3)	χ² = 5.985	0.014

Notes: at-statistic.

Abbreviations: BMI, body mass index.

### Dietary situation

Table 4 details the within-group changes in average daily food intake. After the 12-month follow-up, the intervention group demonstrated comprehensive and significant dietary improvements, including increased intake of beans, nuts, dairy products, vegetables, fruits, poultry, aquatic products, and eggs, alongside significant reductions in refined grains (rice and flour), livestock meat, internal organs, edible oil, and salt (all P < 0.05). While the control group also exhibited some dietary shifts, their intake of key protective foods such as beans, nuts, and aquatic products showed no significant improvement (P > 0.05).

**Table 4:** Comparison of average daily food intake before and after intervention within the two groups (g/d).

Food Category	Group	Before intervention	After intervention	Statistic (t/ Z)	P value
**Rice and its products**	Control	145.60 ± 72.04	126.43 ± 73.22	*t* = 9.878	< 0.001
	Intervention	138.47 ± 58.03	106.00 ± 62.14	*t* = 12.009	< 0.001
**Flour-based products**	Control	176.92 ± 71.16	153.85 ± 74.74	*t* = 12.642	< 0.001
	Intervention	183.90 ± 71.92	128.42 ± 78.56	*t* = 16.166	< 0.001
**Other grains**	Control	29.13 ± 14.88	37.69 ± 14.19	*t* = −4.065	< 0.001
	Intervention	27.26 ± 14.88	39.34 ± 14.28	*t* = −5.864	< 0.001
**Potatoes**	Control	55.17 ± 38.88	55.74 ± 38.36	*t* = −0.775	0.44
	Intervention	57.26 ± 36.41	63.63 ± 36.51	*t* = −10.770	< 0.001
**Total grains and potatoes**	Control	406.82 ± 103.73	373.70 ± 109.27	*t* = 9.906	< 0.001
	Intervention	406.90 ± 100.58	337.39 ± 104.34	*t* = 13.637	< 0.001
**Beans and products**	Control	16.45 ± 11.99	17.10 ± 11.77	*t* = −1.877	0.064
	Intervention	16.00 (7.00, 25.00)	21.00 (9.00, 30.00)	*Z* = −6.383	< 0.001
**Nuts**	Control	5.00 (4.00, 7.00)	5.00 (2.00, 8.00)	*Z* = −0.283	0.778
	Intervention	5.02 ± 2.81	7.16 ± 4.94	*t* = −3.841	< 0.001
**Milk and dairy products**	Control	105.17 ± 64.87	111.43 ± 63.38	*t* = −3.356	0.001
	Intervention	102.15 ± 72.66	136.15 ± 76.72	*t* = −20.620	< 0.001
**Vegetables**	Control	252.75 ± 85.87	272.31 ± 86.00	*t* = −15.189	< 0.001
	Intervention	249.05 ± 88.16	299.58 ± 91.81	*t* = −12.536	< 0.001
**Fruit**	Control	80.00 (60.00, 110.00)	100.00 (70.00, 120.00)	*Z* = −6.943	< 0.001
	Intervention	92.53 ± 30.80	104.60 ± 35.40	*t* = −9.347	< 0.001
**Livestock meat**	Control	53.64 ± 17.00	49.54 ± 16.95	*t* = 10.272	< 0.001
	Intervention	51.57 ± 17.06	39.71 ± 16.28	*t* = 10.102	< 0.001
**Poultry meat**	Control	20.98 ± 10.98	25.32 ± 11.82	*t* = 10.697	< 0.001
	Intervention	19.95 ± 11.62	25.55 ± 12.93	*t* = −6.749	< 0.001
**Total meat**	Control	74.62 ± 20.80	74.86 ± 21.04	*t* = −0.418	0.677
	Intervention	71.52 ± 20.54	65.25 ± 18.97	*t* = 6.062	< 0.001
**Internal organs**	Control	5.00 (2.00, 7.00)	5.00 (2.00, 7.00)	*Z* = −0.030	0.976
	Intervention	3.00 (2.00, 7.00)	2.00 (1.00, 4.00)	*Z* = −6.285	< 0.001
**Aquatic products**	Control	19.21 (7.14, 28.40)	21.30 (14.20, 28.40)	*Z* = −1.553	0.12
	Intervention	21.30 (7.10, 28.40)	47.10 (34.30, 58.60)	*Z* = −8.466	< 0.001
**Eggs**	Control	45.71 ± 21.90	48.63 ± 22.85	*t* = −3.172	0.002
	Intervention	40.00 (30.00, 60.00)	45.00 (30.00, 65.00)	*Z* = −4.634	< 0.001
**Edible oil**	Control	47.25 ± 21.55	39.29 ± 20.13	*t* = 12.553	< 0.001
	Intervention	55.00 (30.00, 65.00)	30.00 (20.00, 40.00)	*Z* = −8.214	< 0.001
**Salt**	Control	9.00 (8.00, 11.00)	8.00 (7.00, 10.00)	*Z* = −5.136	< 0.001
	Intervention	9.00 (7.00, 11.00)	7.00 (6.00, 8.00)	*Z = −7.474*	< 0.001

Notes: Normally distributed data are presented as mean ± standard deviation and analyzed using t-tests. Non-normally distributed data are presented as median (25th percentile, 75th percentile) and analyzed using Z-statistics from Mann-Whitney U tests.

### Physical activity status

As shown in Table 5, the intervention group exhibited significant within-group improvements in leisure activities, household activities, and the overall PASE score after the intervention (all P < 0.05). In contrast, the control group only showed a minor increase in leisure activities, with no significant changes observed in household activities or the PASE total score (both P > 0.05).

**Table 5:** Comparison of PASE scores before and after intervention within the two groups.

Dimension	Group	Before intervention	After intervention	Statistic (t/ Z)	P value
**Leisure activities**	Control	9.64 (4.71, 15.32)	12.18 (5.25, 20.75)	*Z* = −2.946	0.003
	Intervention	11.68 (5.75, 20.36)	18.43 (11.14, 32.04)	*Z* = −5.861	< 0.001
**Household activities**	Control	59.07 ± 27.65	59.63 ± 27.20	*t* = 0.396	0.693
	Intervention	59.27 ± 34.67	63.84 ± 31.94	*t* = −2.016	0.047
**Work-related activities**	Control	0.00 (0.00, 0.00)	0.00 (0.00, 0.00)	*Z* = 0.000	1
	Intervention	0.00 (0.00, 0.00)	0.00 (0.00, 0.00)	*Z* = −0.085	0.933
**PASE Total Score**	Control	84.12 ± 41.86	87.50 ± 42.05	*t* = −1.420	0.159
	Intervention	87.49 ± 47.00	98.69 ± 41.41	*t = −3.869*	< 0.001

Abbreviations: PASE, Physical Activity Scale for the Elderly.

### Changes in clinical indicators

Following the intervention, both groups achieved significant within-group reductions in glycated hemoglobin and LDL-C levels (all P < 0.001), though the intervention group reached lower absolute levels. Notably, regarding renal function, the intervention group showed a significant improvement in creatinine levels (P = 0.002), whereas the control group experienced a significant deterioration in creatinine levels over the 12-month period (P < 0.001; Table 6).

**Table 6:** Comparison of clinical indexes before and after intervention within the two groups.

Clinical Indicator	Group	Before intervention	After intervention	Statistic (t/ Z)	P value
**Glycated hemoglobin (%)**	Control	9.12 ± 0.80	7.99 ± 0.70	*t* = 10.568	< 0.001
	Intervention	8.97 ± 0.70	7.21 ± 0.60	*t* = 17.768	< 0.001
**LDL-C (mmol/L)**	Control	3.44 (2.34, 4.34)	3.20 (2.34, 3.74)	*Z* = −5.450	< 0.001
	Intervention	3.44 (2.34, 4.54)	2.34 (1.90, 3.00)	*Z* = −7.598	< 0.001
**Creatinine (mmol/L)**	Control	77.40 (61.80, 97.70)	92.08 (73.30, 109.90)	*Z* = −4.749	< 0.001
	Intervention	79.81 (62.30, 109.20)	76.86 (61.90, 97.20)	*Z = −3.042*	0.002

Notes: LDL-C, low-density lipoprotein cholesterol.

## Discussion

As the global population continues to age, cardiovascular metabolic complications have emerged as a major global health challenge [19]. With increasing life expectancy, advancements in medical technology, and changes in lifestyle, the elderly have become the primary population affected by cardiovascular metabolic comorbidities, and the presence of multiple conditions has become the norm [20]. Research indicates that cardiovascular metabolic comorbidities not only increase diagnostic and treatment complexity, prolong hospital stays, and exacerbate the consumption of healthcare resources, but also severely impair patients’ quality of life and impose significant economic and social burdens [21]. However, in China, there remains a lack of effective multidisciplinary collaboration in the clinical diagnosis and treatment of cardiovascular metabolic comorbidities [22]. Therefore, how to implement comprehensive management and early intervention has become a key focus of clinical attention. While clinical guidelines strongly recommend comprehensive management for cardiovascular metabolic comorbidities, there is a recognized implementation gap in China due to disjointed primary and tertiary care systems. The novelty of this study lies in testing a pragmatic and scalable pathway that empowers community health workers to execute hospital-formulated comprehensive plans. Our findings demonstrate that this model successfully addresses previously identified gaps in care continuity, yielding superior adherence and metabolic control compared to standard episodic hospital visits. The findings demonstrate that this general practice management model leads to significant improvements in medication adherence, promotes healthier lifestyles, and optimizes key clinical indicators, providing a feasible solution to a major public health challenge.

Metformin, a first-line hypoglycemic drug for patients with type 2 diabetes mellitus (T2DM), is widely recommended by both domestic and international guidelines [23]. Similarly, SGLT2 inhibitors (SGLT2i) and GLP-1 receptor agonists (GLP-1RA) are strongly recommended for patients with T2DM and concomitant ASCVD, given their well-documented cardiovascular benefits [23]. For cardiovascular metabolic comorbidities, aspirin and clopidogrel are essential secondary preventive medications [24,25], whereas statins are first-line cholesterol-lowering agents that significantly reduce the risk of cardiovascular events and all-cause mortality in older adults with diabetes [26,27]. Moreover, ACEI and ARB are the preferred treatments for patients with T2DM and concomitant hypertension as they significantly lower the incidence of major adverse cardiovascular events and cardiovascular and all-cause mortality [23,28–30]. In this study, both groups of patients achieved a high baseline usage rate for these recommended medications. However, after 1 year of follow-up, the intervention group maintained a consistent medication usage rate, whereas the control group showed a clear decline in usage. Several factors could have contributed to drug discontinuation in the control group, including an’ insufficient awareness of patients relating to the importance of medication adherence, concerns relating to side effects, and the financial burden of treatment costs. General practice management addresses these issues by enhancing the understanding of patients with regards to drug therapy via education; this practice can rapidly adjust medications based on individual tolerance, and ensure that patients receive optimized and evidence-based treatments. This holistic approach emphasizes the importance of integrated care in improving outcomes for older adults with cardiovascular metabolic comorbidities. Importantly, the specific components of our intervention were directly mapped to the Chinese Expert Consensus on Comprehensive Management of Cardiovascular Risk. Routine care typically ends with passive and one-way discharge instructions. In contrast, our general practice model actively bridges the hospital-to-community gap. Older patients with multiple comorbidities often suffer from fragmented care when navigating specialized departments. By shifting their continuous monitoring to trained community general practitioners, our model addresses polypharmacy risks, reduces the burden of cross-departmental visits, and provides continuous lifestyle supervision.

In addition to drug therapy, lifestyle interventions play a crucial role in improving outcomes. Obesity, smoking, unhealthy diets and physical inactivity are key modifiable risk factors for cardiovascular and metabolic comorbidities [31]. Excessive body weight and obesity are known to significantly increase the risk of such conditions. Research indicates that losing 5% of body weight can yield notable clinical benefits; however, a 15% loss in weight is known to be optimal. Furthermore, maintaining a BMI within a range of 20.0 to 24.0 kg/m² is recommended [14]. The BMI of patients in our intervention group decreased significantly following intervention, ultimately falling within the suggested range. The cessation of smoking is another pivotal step in reducing the incidence and mortality risk of ASCVD. The risk of ASCVD is known to diminish rapidly after the cessation of smoking and cardiovascular health benefits increase during the period of smoking cessation [32,33]. After one year of management, the number of smokers in the intervention group decreased significantly, thus highlighting the positive impact of targeted interventions.

An unhealthy diet is a well-established global risk factor for cardiovascular and metabolic comorbidities, and its impact is particularly significant and well-documented in China [34,35]. Increasing the consumption of vegetables, eggs and bean products has been shown to prevent and manage T2DM [36], as well as reduce the risk and mortality associated with cardiovascular and cerebrovascular diseases [37]. Reducing the intake of oil, salt, red meat and processed meat, while increasing the consumption of whole grains and beans, has been shown to further reduce the likelihood of developing cardiovascular disease and T2DM [35,38–41]. The dietary habits of patients in the intervention group improved considerably following intervention, and their consumption of high-oil and high-salt foods, refined carbohydrates, and animal-based meat was reduced. Conversely, their intake of vegetables, dairy products, seafood and nuts increased. Although the consumption levels of some food items did not reach the recommended target values, the observed improvements were statistically significant. These represent highly significant changes for the development of a healthier dietary pattern and the mitigation of cardiovascular and metabolic diseases.

The general practice management model has significantly increased the leisure and physical activity time of older adults with cardiovascular metabolic comorbidities. These changes in lifestyle are vital as these can reduce the risk of cardiovascular disease-related mortality [42]. Physical activity provides a potent protective effect against the primary risk factors associated with cardiovascular disease [43]. Existing guidelines recommend that patients with cardiovascular metabolic comorbidities should engage in diverse forms of exercise and incorporate physical activity tailored to their individual conditions in their daily routine [14,15,23,44]. Prior to intervention, no significant differences were observed between our two patient groups with regards to item scores on the PASE scale; this differed from the findings of a previous study in which the total PASE scale scores in a similar patient population were significantly lower than those of healthy older adults [45]. The dual impact of disease and aging causes a steady decline in physical activity in this group of patients. Reduced activity accelerates the progression of disease and exacerbates disease-related limitations, thus creating a self-perpetuating vicious cycle. In this study, addressing these challenges became a central focus of follow-up education. After one year of follow-up, the intervention group achieved significant within-group improvements across multiple physical activity dimensions, including leisure activities, household activities, and the overall PASE score. In contrast, the control group only showed a minor increase in leisure activities. Notably, engagement in leisure activities adhered to the guideline recommendations of ‘low-intensity and moderate-intensity aerobic exercise combined with impedance exercises’ [14,15]. Therefore, the intervention measures described herein led to effective improvements in the physical activity levels of our patients.

By applying standardized treatment and effective disease management combined with the correction of unhealthy lifestyle habits, our patients achieved significantly improvements in several health parameters, including blood sugar, blood lipids and creatinine. A 1% reduction in HbA1c can reduce the risk of coronary heart disease events by 15% [46,47]. For older adults with cardiovascular metabolic comorbidities, the HbA1c control target is set to < 8% [14]. Following the intervention, patients in the intervention group successfully met this HbA1c target through significant within-group reductions. In addition, the significant within-group reduction of LDL-C levels represents the primary therapeutic target for minimizing ASCVD risk. Most notably, our longitudinal analysis revealed a significant renal protective effect: while creatinine levels in the control group significantly deteriorated over the 12-month period, the intervention group demonstrated significant improvements. This stark contrast underscores the critical value of continuous holistic management in delaying target organ damage. In this study, the intervention group achieved significant improvements in LDL-C levels following intervention; however, not all patients achieved the target levels. This discrepancy can be attributed to the high rate of statin use; this is because some patients likely required combination therapy to achieve optimal blood lipid levels. Extended, long-term intervention may further enhance LDL-C outcomes.

Although we did not conduct a formal health economic evaluation, the significant improvements in HbA1c, LDL-C, and medication adherence observed in our study are established clinical proxies for reduced long-term cardiovascular events and hospital readmissions. Furthermore, utilizing existing community health service centers for continuous follow-up is inherently a resource-efficient approach compared to requiring elderly patients to make frequent visits to tertiary hospitals. It optimizes medical resource distribution and reduces out-of-pocket and travel burdens for patients.

There are some limitations to this study that need to be considered. First, a formal a priori statistical power analysis was not conducted. The sample size of 200 patients was determined empirically based on our clinical experience, historical patient volume in our General Medical Department, and the practical follow-up capacity of the collaborating community health centers. Nevertheless, post-hoc evaluation indicates that this sample size was sufficient to detect statistically and clinically significant differences in our primary clinical indicators. Second, the single-center design and short follow-up duration limit the generalizability of our findings. To validate the efficacy of the general practice management model, future studies involving larger sample sizes and extended follow-up periods are now warranted.

## Conclusion

In summary, the general practice management model significantly enhanced medication adherence in a cohort of older adults with cardiovascular metabolic comorbidities. This management model effectively addressed unhealthy lifestyle habits and outcomes, such as obesity, smoking, poor dietary choices, and insufficient physical activity, thus demonstrating its practical importance for the management of risk factors associated with ASCVD. Furthermore, comprehensive management improved clinical indicators, providing robust support for the prevention and treatment of cardiovascular metabolic comorbidities in older adults.

## Supporting information

S1 CONSORT Checklist CONSORT checklist of information to include when reporting a randomised trial. (DOCX)
